# Regulation of Human Stem Cells by Functional Food Components: How Vitamins, Minerals and Phytochemicals Influence Mesenchymal Stem Cells’ Fate and Function

**DOI:** 10.3390/nu17223548

**Published:** 2025-11-13

**Authors:** Marta Kot, Patrycja Bronowicka-Adamska, Malgorzata Tyszka-Czochara

**Affiliations:** 1 Department of Transplantation, Faculty of Medicine, Institute of Pediatrics, Jagiellonian University Medical College, Wielicka 265, 30-663 Krakow, Poland; marta.kot@uj.edu.pl; 2 Department of Medical Biochemistry, Faculty of Medicine, Jagiellonian University Medical College, 7c Kopernika, 31-034 Krakow, Poland; patrycja.bronowicka-adamska@uj.edu.pl; 3 Faculty of Pharmacy, Jagiellonian University Medical College, Medyczna 9, 30-688 Krakow, Poland

**Keywords:** stem cells, vitamins, minerals, phytochemicals, nutritional regulation, differentiation, tissue regeneration

## Abstract

Mesenchymal stem cells (MSCs) are multipotent cells capable of self-renewal and differentiation into specialized cell types, which play an important role in maintaining homeostasis and tissue regeneration in humans. The effectiveness of MSCs depends largely on their immunomodulatory properties and ability to regenerate damaged tissues. Biological activity of MSCs is modulated by environmental factors, including dietary components such as vitamins, minerals, and phytochemicals which influence their proliferation, aging, inflammatory response and resistance to oxidative stress. The article aims to highlight the importance of micronutrients and phytochemicals in modulating the MSCs’ performance and therapeutic potential, with a focus on the role of bioactive food components in regulating metabolism, regenerative efficacy and protective mechanisms of stem cells. Vitamins and trace elements are essential for antioxidant protection by eliminating reactive oxygen species, maintaining mitochondrial function and preserving cell viability under stressful conditions. Micronutrients and phytochemicals can modulate the immunomodulatory activity of MSCs by altering the cytokine secretion profile, reducing pro-inflammatory mediators while enhancing anti-inflammatory factors. However, both deficiency and excessively high concentrations of natural compounds can impair stem cell function. Interdisciplinary knowledge about the impact of micronutrients on the functioning of mesenchymal stem cells creates new opportunities in personalized medicine and nutrition. Understanding the mechanisms regulating MSCs activity under the influence of diet components may contribute to the development of individualized therapeutic strategies aimed at supporting tissue regeneration, delaying aging processes, and improving the prevention and treatment of chronic diseases. This knowledge is applicable in the design of functional foods and dietary supplements, making it particularly valuable for specialists in personalized nutrition and functional food development.

## 1. Introduction

**Figure 1:**
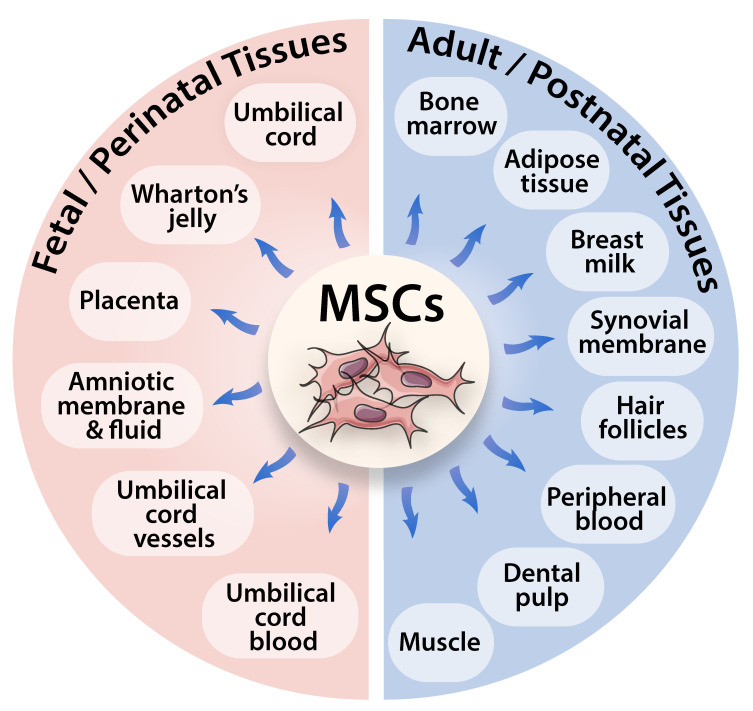
Overview of perinatal and adult tissue sources of MSCs. This schematic representation shows the diverse origins of mesenchymal stem cells. Perinatal sources include birth-associated tissues like the placenta, umbilical cord, and amniotic membrane. Adult sources are found in postnatal tissues such as bone marrow, dental pulp, and peripheral blood.

Stem cells are undifferentiated cells capable of extensive self-renewal and differentiation into specialized tissues, making them essential for organismal development, maintenance, and repair [1,2,3]. Increasing scientific interest has been directed toward mesenchymal stem cells (MSCs). These are a type of adult stem cell that persists throughout all stages of life and is widely distributed across various tissues in the human body (Figure 1) [2]. Due to their relatively high abundance in the body and their regenerative capacity, bold efforts are being made to utilize mesenchymal stem cells in regenerative medicine to restore the function of injured tissues and potentially counteract age-related degeneration [4,5]. In humans, impaired stem cell function is linked to aging and numerous chronic diseases, as the decline of regenerative capacity of the cells limits the body’s ability to recover from damage [1]. Thus, understanding how to preserve and even enhance these stem cells’ function is of great interest for promoting human health and longevity.

Emerging evidence indicates that dietary factors and nutritional status can influence stem cell performance in human body [2,6,7,8,9,10]. Micronutrients not only influence cellular metabolism but also are important signaling molecules that can modulate regulatory pathways and determine stem cell activity. In particular, vitamins, minerals and phytochemicals are of critical importance in maintaining stem cell proliferation and self-renewal by influencing gene expression and epigenetic regulation [7,8,9,11,12,13,14]. Growing evidence indicate that nutritional regulation is an emerging target for stem cell function, which could affect not only cell survival but also stem cell pools in tissues. Recent findings indicate a direct link between the action of specific molecules and stem cell–mediated tissue homeostasis and differentiation. Vitamins A, D, C, E, the B vitamins, and minerals such as zinc, selenium, magnesium, and calcium have been highlighted as key micronutrients for stem cell function [13,15,16,17].

Vitamins and minerals can be delivered to human body from natural sources (e.g., fruits, vegetables, nuts, oils, dairy, meat). However, properly designed fortified products may provide selected micronutrient(-s), also in higher concentration than found in foods, targeting specific effects on cellular level [2]. Therefore, the concept of functional foods that provide health benefits beyond basic nutrition is particularly relevant to stem cell biology. Functional food components have been demonstrated to have numerous physiological benefits in humans by improving health or reducing chronic diseases risk [18]. Health-promoting properties of functional foods are mediated by interactions of their components with signaling pathways and gene expression networks. Because of this, functional food micronutrients may be plausible modulators of stem cell function, potentially enhancing regenerative outcomes and protecting stem cells from stress [19]. In this review, we provide a comprehensive analysis of how vitamins, minerals and phytochemicals influence the regulation of mesenchymal stem cells. We place particular emphasis on their multifaceted roles in promoting proliferation, self-renewal, and differentiation, controlling senescence, tissue regeneration and remodeling, immunoregulation, and maintaining redox homeostasis. Furthermore, we explore their indispensable contribution to epigenetic regulation and the cellular adaptation to stress. Within this context, we especially examine the roles of vitamins and essential minerals in regulating the biology of mesenchymal stem cells, which are critical for the body’s renewal processes.

### Embryonic Stem Cells vs. Adult Stem Cells

**Figure 2:**
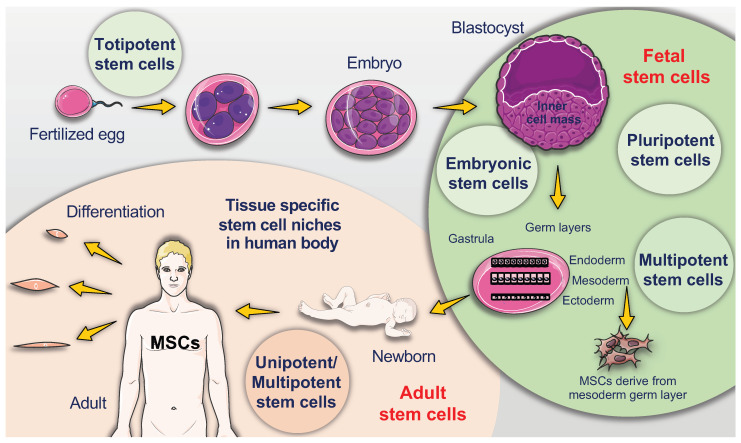
Developmental hierarchy and sources of stem cells. The figure illustrates the origins and differentiation potential of various stem cell types. The process begins with pluripotent embryonic stem cells (ESCs) derived from the inner cell mass of the blastocyst. These cells can differentiate into the three primary germ layers (ectoderm, mesoderm, and endoderm) during the gastrulation stage. Mesenchymal stem cells (MSCs) are multipotent cells that originate from the mesodermal layer. In contrast to embryonic sources, there are also postnatal sources, including adult (somatic) stem cells residing in tissue-specific niches and perinatal stem cells isolated from the umbilical cord of a newborn.

Stem cells can proliferate and differentiate into a variety of cells in human body. Early embryo cells (2- to 4-cell stage blastomeres) are totipotent and capable of differentiating into every cell type in a new organism (Figure 2). Embryonic stem cells (ESCs), derived from the inner cell mass of the blastocyst, are pluripotent. This means they are capable of self-renewal and can differentiate into all three primary germ layers (ectoderm, mesoderm, and endoderm), thereby forming any cell type of the body, but not the extra-embryonic tissues like the placenta [1,20]. Adult (somatic) stem cells are defined by their ability to differentiate into tissue-specific cells, have a limited self-renewal capability (they can be multipotent or unipotent) and can regenerate selected tissues. Due to their therapeutic potential, they have recently gained significant interest from researchers, and many attempts are being made to use them in clinical practice [4]. Therefore, much attention is currently being paid to study environmental and internal factors that may influence the function of these cells.

Adult stem cells remain active throughout the human lifespan because their pool can be replenished through a specific type of cell division—asymmetric division. This process ensures that one daughter cell retains the properties of the original stem cell, thereby maintaining the stem cell population. Therefore, stem cells play a particularly vital role in tissue renewal. In the human body, adult stem cells reside in specific microenvironments known as niches, which, as recent studies have shown, regulate their activity and fate. Moreover, nutrient availability is one of the most important factors that shape stem cells microenvironment [21,22].

Mesenchymal stem cells, among other types of stem cells, deserve particular attention due to their high self-renewal capacity and their beneficial effects in human body. MSCs acting through multiple mechanisms exhibit remarkable regenerative potential and contribute to tissue repair, immunomodulation and homeostasis [4,23]. In this review, we focus on the processes through which micronutrients may influence the functions of MSCs.

## 2. Materials and Methods

**Scheme 1:**
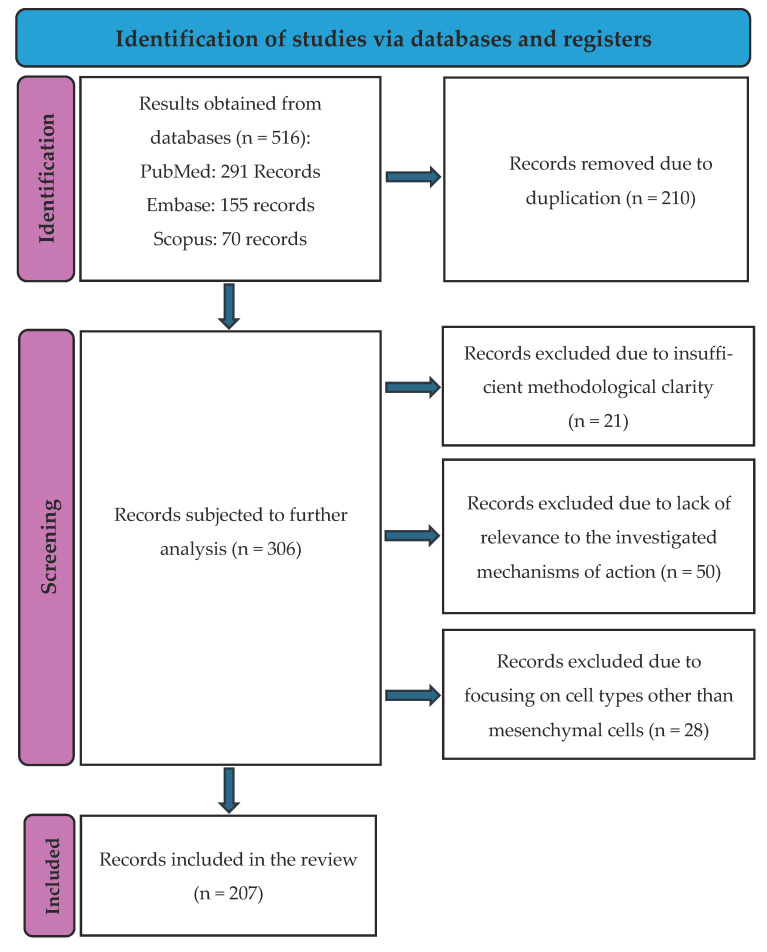
Diagram illustrating the selection process of scientific publications for the literature review. Based on https://www.prisma-statement.org/prisma-2020, accessed on 18 October 2025.

The bibliography included in this review was compiled because of a comprehensive search of the PubMed/MEDLINE, Embase, and Scopus databases. The strategy focused on original research articles and review papers by using keywords such as mesenchymal stem cells, human stem cells, stem cell differentiation, stem cell proliferation, MSC function, stem cell nutrition regulation, functional foods, bioactive compounds, phytochemicals, nutraceuticals, vitamins, minerals, polyphenols, antioxidants, flavonoids, dietary supplements, oxidative stress, inflammation, epigenetic regulation, signaling pathways in MSCs, Wnt signaling and MSCs, MAPK pathway, PI3K/Akt, autophagy, mitochondrial function in MSCs, regenerative medicine, tissue engineering, aging and stem cells, bone regeneration, adipogenesis/osteogenesis, immunomodulation by MSCs. A block search strategy was used with the use of the Boolean operators “AND” and “OR”. Following searching of the database, duplicate records were removed. A rigorous selection procedure was then carried out, excluding papers according to three main criteria: unclear or inadequately reported research methodology, unimportance to the mechanisms of action investigated, and research conducted on stem cell types other than mesenchymal stem cells, which were the subject of this review. This stringent screening process yielded the final set of studies covered in this paper. The selection process is illustrated in the PRISMA diagram (Scheme 1).

## 3. Therapeutic Properties of Mesenchymal Stem Cells

MSCs, besides their ability to self-renew, are characterized by a capacity to differentiate into a variety of cell types, particularly those of mesodermal origin, such as osteoblasts (bone cells), adipocytes (fat cells), and chondrocytes (cartilage cells) [24,25]. Consistent with this, MSCs are distributed throughout the body in tissues such as bone marrow, adipose tissue, dental pulp (Figure 1) [25,26,27,28]. In particular, perinatal tissues such as the placenta, umbilical cord, Wharton’s jelly, and amniotic fluid are rich in highly potent stem cells. They demonstrate a strong capacity to differentiate into several cell lineages, including muscle, neural, chondrogenic, bone and adipose cells [2,3,5,7,17,29,30,31,32,33]. Recently, it was reported that human breast milk may serve not only as a nutrition supply, but it may also contain a relatively large population of MSCs [34]. These cells display stem cell properties and differentiate into various lineages in response to stimulation by microenvironmental factors. The cells isolated from fresh breastmilk express typical MSC markers (CD90, CD105, CD73) [35] and the cell population depends on, among others, the nutritional status of the mother and baby. It has been shown that the cells cultured from human breast milk can secrete molecules typical of adult MSCs, such as growth factors (vascular endothelial growth factor and hepatocyte growth factor), which are essential for the proliferation, migration, and angiogenesis of stem cells and other cells in the body [36]. Further studies revealed that milk derived-MSCs may be involved in the development of tissues and organs in the infant. Abd Allah SH et al. demonstrated in rabbits that orally administered MSCs pass through the gastrointestinal tract mucosa, integrate into the organs of offspring and even have the potential to proliferate in situ [37]. Nevertheless, more studies are needed to determine the functional benefits of milk-derived MSCs, whether they may cause an increase in cell proliferation and growth of newborn tissues. Recent in vitro experiments have provided interesting results showing that MSCs isolated from human breast milk can differentiate into neural lineages [38]. This highlights that breast milk is a source of stem cells with high differentiation potential and is available without any ethical concern.

One of the most important features of MSCs are their immunomodulatory properties that can influence the activity of various immune cell types, in particular, promoting the polarization of macrophages from a pro-inflammatory (M1) to an anti-inflammatory (M2) phenotype, and enhancing regulatory T cell populations [39,40,41,42]. This makes MSCs particularly useful in conditions of inflammation developing during tissue damage. As mentioned, MSCs secrete an array of bioactive factors, including cytokines and growth factors, which not only support tissue repair and regeneration but also modulate the local immune response [43,44]. Their regenerative capacity is enhanced by their involvement in angiogenesis, the process of new blood vessel formation, which is vital for supplying injured tissues with nutrients and oxygen [45]. Studies have demonstrated that MSCs can promote wound healing also by facilitating cellular communication and extracellular matrix remodeling [43,46]. Their ability to migrate to sites of injury even from distant organs and capacity to exert paracrine effects in situ underlines their potential particularly to counteract chronic diseases and degenerative conditions [47,48].

Growing body of evidence shows that vitamins and minerals are important modulators of MSCs function, playing essential roles in proliferation, differentiation, and cellular homeostasis [8,9,17,33,49,50,51]. The interplay between MSCs and micronutrients present in the microenvironment is increasingly recognized as a key determinant of cell fate. Within a particular niche, the microenvironment can be modulated to maintain stem cells in an undifferentiated state (preserving their stemness) and retain their potential to transform into defined cell types. Alternatively, the differentiation processes can be directed toward specific cell types needed to counteract a particular disorder.

## 4. Vitamins and Trace Elements Shape MSCs Fate—Regulation of Proliferation, Self-Renewal, and Senescence in Mesenchymal Stem Cells

### 4.1. The Influence of Vitamins on MSC Fate

The therapeutic utility of mesenchymal stem cells is intrinsically linked to their capacity for robust proliferation, effective self-renewal, and resistance to cellular senescence. A growing body of evidence indicates that various vitamins and minerals are key modulators of these cellular processes (Table 1) [17]. Their influence is often characterized by a dose-dependent and context-specific nature, highlighting the necessity for precise optimization in both clinical and in vitro settings. Among the vitamins, vitamin C (ascorbic acid) is a well-established promoter of MSC proliferation and self-renewal [52,53]. This vitamin can accelerate cell population doubling and upregulate the expression of pluripotency-related genes, thereby preserving the stemness of MSCs [8,54]. Conversely, excessive concentrations can inhibit cell growth and may even induce apoptosis [8]. Furthermore, vitamin C contributes to delaying cellular senescence, partly by supporting telomere maintenance and modulating key signaling pathways associated with aging [55,56,57]. Vitamin D (1,25-dihydroxyvitamin D_3_) also exerts a complex, modulatory influence on MSCs fate. Its active metabolite has been reported to support the maintenance of stemness by enhancing the expression of pluripotency markers while concurrently reducing the prevalence of senescent cells [58,59]. The effects of vitamin D are markedly dose-dependent, both deficiency and excessive supplementation lead to the suppression of MSC proliferation [60]. It has also been implicated in improving MSC resistance to metabolic stress-induced dysfunction [61]. Other lipid soluble vitamins including vitamin E also play a regulatory role in MSCs growth regulation. It can act synergistically with vitamin D to modulate proliferation and influences signaling pathways related to cell survival [9,54,62]. The B-group vitamins are fundamental as cofactors enzymes catalyzing the core metabolic pathways. Notably, vitamins B6, B9, and B12 are integral to one-carbon metabolism, thereby influencing epigenetic mechanisms such as DNA and histone methylation and therefore are crucial for self-renewal processes and lineage commitment [63,64]. Furthermore, nicotinamide (vitamin B3) has been shown to delay replicative senescence in MSCs [65].

### 4.2. The Role of Trace Elements in MSC Maintenance

In addition to vitamins, several trace elements are critical for maintaining the functional integrity of MSCs. Magnesium, for instance, has been demonstrated to promote MSCs viability and proliferation, with dose-dependent effect [66,67,68,69]. Magnesium deficiency can accelerate cellular aging and stem cell exhaustion, while excessively high concentrations can be inhibitory or even cytotoxic, as presented in Table 1 [70]. Similarly, zinc is crucial for MSCs maintenance, as optimal levels support proliferation and enhance the expression of key pluripotency and telomerase-related genes, whereas high concentrations are cytotoxic [10]. Selenium also shows a double effect, supporting MSC viability and mitigating senescence at low concentrations, but becoming detrimental at higher levels [49,69]. The impact of iron is also highly dependent on its concentration and chemical form [71,72]. Lastly, there are experimental evidence that calcium, particularly when delivered through specific compounds, can effectively promote the proliferation of various MSCs populations [73,74,75,76].

**Table 1:** The influence of vitamins and trace elements in MSCs fate and maintenance.

Substance	Effect on MSCs	Concentration	Cell Type/Model	Refs.
**Vitamin C**	↑ Proliferation, self-renewal, expression of pluripotency genes (Nanog, Oct4, Sox2) ↓ Senescence (reduced SA-β-gal+ cells, p16 suppression) Growth inhibition/apoptosis at higher doses.	10–250 µM (stimulation), >250 µM (inhibition), 200 µM (anti-aging effect)	Gingival stem cells (GSCs); Rat MSCs	[8,56]
**Vitamin D**	↑ Expression of pluripotency genes (Nanog, Sox2, Oct4), resistance to dysfunction ↓ Number of senescent cells, p16 expression Proliferation suppression at deficient and excessive doses.	1 × 10^−7^ M (with Vit. E) 0 IU/kg or 1000 IU/kg (suppression), 250–500 IU/kg (optimal)	Bone marrow-derived MSCs (BM-MSCs); Rat lung-derived MSCs (in vivo model)	[17,54,58,60,61]
**Vitamin E**	↓ Apoptosis (via AKT pathway modulation) ↑ Expression of proliferative markers ↓ Proliferation (in combination with Vit. D)	12 µM (with vit. D)	Human dental pulp stem cells (hDPSCs); Gingiva-derived stem cells (GSCs)	[9,77,78,79]
**Vitamin B3**	↑ MSC survival ↓ Delays replicative senescence	5 mM	Human BM-MSCs	[65]
**Magnesium (Mg^2+^)**	↑ Viability, proliferation, adhesion. Inhibition/cytotoxicity at high concentrations.	1.2–1.8 mM (MgCl_2_), 5.7–8.7 mM (Mg^2+^), >20 mM (MgSO_4_) (inhibition), 32.1 mM (cytotoxicity)	Human MSCs; Rat bone marrow MSCs	
**Zinc (Zn^2+^)**	↑ Proliferation, expression of pluripotency genes (Oct4, Sox2, Nanog) and telomerase (TERT). Cytotoxicity at higher concentrations.	20 µM (optimal) >100 µM (cytotoxicity)	Umbilical cord-derived MSCs (hUC-MSCs)	[10]
**Selenium (Se)**	↑ Viability ↓ Senescence. Reduced viability at higher concentrations	50 ng/mL (Se nanoparticles) 0.1 µM (sodium selenite), 300 ng/mL (reduced viability)	Rat bone marrow MSCs	[49,69]
**Iron (Fe)**	↑ Proliferation (as FeSO_4_) ↓ Proliferation and senescence (as iron oxide nanoparticles)	4 × 10^−8^ M (FeSO_4_), 100 µg/mL (nanoparticles)	Adipose-derived MSCs; BM-MSCs	[71,72]
**Calcium (Ca^2+^)**	↑ Proliferation/survival	0.01–100 mg/mL (stimulation), 1 mg/mL (optimal), 4–10 mM (stimulation), 6 mM (optimal), >6 mM (inhibition); As calcium phosphate nanoparticle—not specified	**In vitro**: Human stem cells of the apical papilla (SCAPs); BM-MSCs	[73,75,76,80]

Abbreviations: AKT—Protein kinase B, BM-MSCs—bone marrow-derived mesenchymal stem cells, GSCs—gingival stem cells, hDPSCs—human dental pulp stem cells, hUC-MSCs—human umbilical cord-derived mesenchymal stem cells, MSCs—mesenchymal stem cells, Oct4—octamer-binding transcription factor 4 (POU5F1), SA-β-gal—senescence-associated beta-galactosidase, SCAPs—stem cells of the apical papilla, Sox2—SRY (sex-determining region Y)-box 2, TERT—telomerase reverse transcriptase, the arrow upwards (↑) indicates stimulation or an increase, the arrow downwards (↓) indicates inhibition or a decrease.

## 5. Modulation and Control of MSC Differentiation by Vitamins and Minerals

The differentiation of mesenchymal stem cells can be effectively modulated by a range of vitamins and minerals. As discussed below, micronutrients may preferentially direct MSCs toward the osteogenic lineage while concurrently suppressing adipogenesis and chondrogenesis, thereby playing a critical role in tissue homeostasis and regenerative applications [49,50,57,73,81,82,83] (Figure 2 and Table 2).

### 5.1. Modulation of Osteogenesis

In cell cultures, ascorbic acid (vitamin C) is a key component of osteogenic differentiation media, essential for promoting the osteogenic potential of MSCs. It supports collagen synthesis and upregulates the master transcription factor Runx2, which in turn drives the expression of downstream osteogenic genes [32,57,84,85]. Likewise, 1,25-dihydroxyvitamin D_3_ robustly drives MSCs toward osteogenesis by activating vitamin D receptor (VDR). This activation enhances the expression of key osteogenic markers and simultaneously inhibits adipogenic differentiation [49,61]. The pro-osteogenic effects of vitamin D are strengthened by co-treatment with growth factors or dexamethasone and are mediated through signaling pathways such as Wnt/β-catenin and BMP2 [49,61,86,87,88,89,90,91,92,93]. Vitamin E and vitamin K_2_ also contribute to osteogenesis, often acting synergistically with vitamin D to promote the differentiation of MSCs towards the osteogenic lineage and enhance the expression of key osteogenic transcription factors [9]. Furthermore, calcium is a recognized promoter of osteogenic differentiation, with calcium deposition and alkaline phosphatase (ALP) activity serving as key indicators of osteoblastic maturation [70,94].

### 5.2. Regulation of Adipogenesis and Chondrogenesis

Reflecting this dual function, several vitamins that promote osteogenesis concurrently suppress adipogenic differentiation, thereby maintaining a crucial balance in skeletal tissue homeostasis [50,95]. Vitamin D_3_, for instance, exerts a dose-dependent anti-adipogenic effect by reducing lipid accumulation and downregulating the expression of adipocyte-specific genes, a mechanism involving the inhibition of the transcription factor PPARγ2 [50,95,96,97]. Similarly, vitamin C typically inhibits adipogenesis in MSCs but can, under certain conditions, preserve their multilineage potential [8,81]. Vitamin C was demonstrated to enhance chondrogenic differentiation and protect chondrocytes from oxidative stress [98,99].

### 5.3. Influence of Inorganic Ions on MSC Differentiation

Magnesium (Mg^2+^) is a critical regulator of bone formation, where elevated extracellular concentrations enhance osteogenic differentiation through the activation of signaling pathways, in particular mediated by Notch and Wnt proteins, as presented in Table 2 [67,100,101,102,103]. However, its role appears to be biphasic, as excessively high concentrations may impair late-stage matrix mineralization [68]. Magnesium also promotes chondrogenesis by supporting the synthesis of cartilage-specific extracellular matrix [67,104,105,106]. Other inorganic ions also strongly promote osteogenesis. Calcium and phosphate ions, often delivered via mineralized matrices, drive the expression of key osteogenic markers through various signaling pathways [73,107,108]. Zinc ions enhance osteoblast differentiation and matrix mineralization, and the effect was particularly observed in cell cultures grown on scaffolds with element incorporated to the structure [73,109]. Finally, selenium has been shown to shift MSCs’ fate toward the osteogenic lineage while simultaneously inhibiting adipogenesis [49].

**Table 2:** Modulation and control of MSC differentiation by vitamins and inorganic ions.

Substance	Differentiation	Effect and Mechanism	Concentration	Cell Type/Model	Refs.
**Vitamin C**	Osteogenesis	↑ Restores osteogenic potential in senescent MSCs by activating telomerase. Enhances mineralization and expression of RUNX2, ALP, COL1A1. Essential for collagen synthesis, which in turn upregulates Runx2	50 µg/mL	Senescent human BM-MSCs; ASCs	[32,57,85]
	Adipogenesis	↓ Suppresses differentiation into adipocytes	250–500 μM, 150 μM (optimal)	Mouse embryonic mesoderm-derived mesenchymal cells	[81]
	Chondrogenesis	↑ Enhances differentiation and protects chondrocytes from oxidative stress (H_2_O_2_)	50 μg/mL (~170 μM) (ADSCs); 50 μmol/L (GSCs)	Glial-derived stem cells (GSCs); Adipose-derived MSCs (ADSCs)	[8,98]
**Vitamin D**	Osteogenesis	↑ Enhances expression of ALP, OCN, RUNX2 via the vitamin D receptor (VDR). Synergizes with BMP-2, TGF-β1, metformin, and dexamethasone. Activates Wnt/β-catenin and BMP2 signaling. Reduces ROS	20 nM (peak effect)	Bone marrow MSCs (BM-MSCs)	[49,50,61,87,89,92,93]
	Adipogenesis	↓ Reduces lipid accumulation. Downregulates adipocyte-specific genes by inhibiting the transcription factor PPARγ2	20 nM (strongest inhibition)	Human BM-MSCs	[50,95,96,97]
**Vitamin E**	Osteogenesis	↑ Enhances osteogenic commitment, particularly in combination with vitamin D (0.1 μM)	12 μM (hDPSCs); 6.06 μg/mL α-tocoferol (concentration in human serum used in BM-MSCs culture)	Human dental pulp stem cells (hDPSCs); BM-MSCs	[9,82]
**Vitamin K_2_**	Osteogenesis	↑ Synergistically with vitamin D_3_ (10 nM) enhances the expression of osteocalcin, osterix, and Runx2	10 nM	Diet-induced obese mouse models (primary osteoblasts)	[110]
**Magnesium**	Osteogenesis	↑ Enhances early differentiation via Notch and Wnt signaling and autophagy. High concentrations may impair late-stage mineralization	5–10 mM (enhances) <1.3 mM (favors) 0.1 mM (stimulates autophagy)	Rat and human BM-MSCs	[67,68,100,101,102,103]
	Chondrogenesis	↑ Supports differentiation and synthesis of cartilage-specific extracellular matrix via integrin-mediated signaling	~5 mM	BM-MSCs; Synovial MSCs	[104]
**Calcium**	Osteogenesis	↑ Upregulates OCN, BSP, ALP expression. Mediated through L-type calcium channels and CaMKII signaling	Not specified—used as calcium phosphate biomaterials surface	Human BM-MSCs; hMSC immunoselected with STRO1 antibody; rabbit BM-MSC	[111,112,113]
**Zinc**	Osteogenesis	↑ Enhances osteoblast differentiation, matrix formation, and mineralization	Not specified—used as MOF nanoparticles	Adipose-derived MSCs	[109]
**Selenium**	Osteogenesis	↑ Shifts MSC fate towards osteogenic lineage. Increases ALP activity and osteogenic gene transcription	25–100 ng/mL (50 ng/mL optimal)	hESC-derived MSCs	[49]
	Adipogenesis	↓ Suppresses adipocyte differentiation	25–100 ng/mL	hESC-derived MSCs	[49]

Abbreviations: ADSCs, ASCs—adipose-derived mesenchymal stem cells, ALP—alkaline phosphatase, BM-MSCs—bone marrow-derived mesenchymal stem cells, BMP-2—bone morphogenetic protein 2, BSP—bone sialoprotein, CaMKII—calcium/calmodulin-dependent protein kinase II, COL1A1—collagen type I alpha 1 chain, GSCs—glial-derived stem cells, hDPSCs—human dental pulp stem cells, hESC—human embryonic stem cells, MOF—metal–organic framework, MSCs—mesenchymal stem cells, Notch—Notch signaling pathway, OCN—osteocalcin, PPARγ2—peroxisome proliferator-activated receptor gamma 2, ROS—reactive oxygen species, RUNX2—runt-related transcription factor 2, STRO1—stromal precursor antigen-1, TGF-β1—transforming growth factor-beta 1, VDR—vitamin D receptor, Wnt—Wnt signaling pathway, the arrow upwards (↑) indicates stimulation or an increase, the arrow downwards (↓) indicates inhibition or a decrease.

## 6. Vitamins and Micronutrients in MSC-Mediated Tissue Regeneration and Remodeling

The capacity of mesenchymal stem cells to facilitate tissue repair is significantly influenced by the local biochemical environment, with various vitamins and minerals playing a direct role in modulating their regenerative functions.

### 6.1. Vitamins Enhancing Tissue Regeneration

Among the vitamins, the role of vitamin C in tissue remodeling is particularly well-established (Table 3). As an enzyme cofactor for critical importance in collagen synthesis, it strongly supports extracellular matrix (ECM) formation, which is essential for tissue integrity and the regeneration of bone defects [32,114]. Vitamin C has also been shown to improve neovascularization and overall tissue repair in vivo [115]. Similarly, vitamin D directly enhances the regenerative potential of MSCs. It promotes MSC-mediated bone regeneration by directing osteogenic differentiation and modulating the local immune environment [50,116]. Furthermore, it contributes to vascular repair and angiogenesis by improving the function of endothelial progenitor cells and has been shown to support proper lung development in perinatal models [60,117]. In vitro studies shown that MSCs pretreatment with vitamin E protects the cells from oxidative stress-induced damage, thereby enhancing their survival and preserving regenerative functionality [77]. These data are promising for cartilage regeneration, since vitamin E-treated MSCs promote chondrogenic differentiation and may slow the progression of osteoarthritis by inhibiting apoptosis and senescence [118,119]. In contrast to the supportive roles of other vitamins, it is noteworthy that treatment with vitamin B6 has been observed to accelerate the clearance of administered MSCs from the body in a murine model. These phenomena should be thoughtfully considered in therapeutic applications [120].

### 6.2. Mineral Compounds in MSC-Mediated Tissue Regeneration

Beyond vitamins, essential minerals are also integral to MSC-mediated repair. Magnesium is crucial for both bone homeostasis and cartilage regeneration. According to Shimaya et al. (2010) it enhances the adhesion of synovial MSCs to injured sites, promotes cartilage matrix synthesis, and induces apatite crystal growth on surfaces, a key step in in vivo bone formation and remodeling [104,121]. Likewise, zinc improves the therapeutic efficacy of MSCs by enhancing their migration and adhesion, which are critical processes for homing to sites of injury [10]. Zinc-containing biomaterials have been engineered to simultaneously promote osteogenesis and angiogenesis, thereby supporting complex tissue repair [109]. It was shown that iron delivered via iron oxide nanoparticles (IONPs) enhances the migration of MSCs to injury sites. This improved homing facilitates wound healing and angiogenesis, and IONP-labeled MSCs have also been successfully used to improve bone repair and reduce liver fibrosis in preclinical models [122,123,124].

**Table 3:** The vitamins and micronutrients in MSC-mediated tissue regeneration and remodeling.

Substance	Tissue Effect	Mechanism	Dose	Cell Type/Model	Refs.
**Vitamin C**	**↑** Enhances bone defect regeneration and tissue repair	Acts as a cofactor for collagen synthesis; supports extracellular matrix (ECM) formation and deposition; improves blood perfusion and neovascularization.	200 μM (cell culture media component); 50 μg/mL (regeneration/tissue engineering); 0.05–0.2 mM (collagen synthesis cofactor)	**In vivo** ischemic limb models in mice (SCB-MSCs); ASCs	[13,32,55,71,91,94,114,115,125,126,127,128]
**Vitamin D**	**↑** Promotes bone regeneration, vascular repair, and lung development	Promotes osteogenic differentiation; modulates the immune environment; improves endothelial progenitor cell (EPC) adhesion and migration; alleviates TNF-α-mediated inflammation.	250 and 500 IU/kg (optimal dietary doses)	Rat models (Perinatal dietary supplementation); lung MSCs	[50,60,116,117]
**Vitamin E**	**↑** Enhances MSC survival and cartilage regeneration	Preconditioning protects MSCs from oxidative stress (H_2_O_2_); upregulates TGF-β; downregulates apoptosis genes; reduces VEGF and LDH release; increases proteoglycan content in cartilage.	100 μM	**In vitro** preconditioning of MSCs; **In vivo:** osteoarthritis rat models.	[77,118,119]
**Vitamin B6**	**↑** Accelerates clearance of MSCs from the body	Led to lower numbers of detectable ucMSCs 4 h post-injection, especially when combined with retinoic acid.	1 μM	Murine model of hepatitis (mice treated with human ucMSCs)	[120]
**Magnesium**	**↑** Supports bone homeostasis and cartilage regeneration	Enhances MSC adhesion to defects; promotes cartilage matrix synthesis; induces apatite crystal growth on scaffolds for mineralization.	5 mM MgCl_2_ (in PBS)— in vivo rabbit model; 1 mM and 10 mM MgCl_2_ (in PBS)— ex vivo studies; 5 mM and 10 mM MgCl_2_ (in PBS)— in vitro studies. Not specified (used as Mg-BCP micro-scaffolds prepared in terms of 0.01 Mg/Ca and 1.602 of (Ca+Mg)/P mole ratios) (human AT-MSCs).	**In vivo***:* using a rabbit osteochondral defect model (rabbit synovial MSCs); **Ex vivo*:*** with osteochondral tissue (human synovial MSCs); **In vitro**: chondrogenesis assessment, cell adhesion (human synovial MSCs); **In vitro*:*** studies with human AT-MSCs.	[104,121]
**Zinc**	**↑** Promotes tissue repair by enhancing cell homing	Significantly improves MSC migration and adhesion; scaffolds co-doped with copper promote both osteogenesis and angiogenesis.	5–100 μM (stimulation), 20 μM (optimal), >250 μM (inhibition); Zinc-containing scaffolds: PLLA@MOF containing 0.30 mol of ZnO	**In vitro** scratch assays (hUC-MSCs); Human adipose tissue-derived MSCs	[10,109]
**Iron (IONPs)**	**↑** Improves wound healing, and reduces liver fibrosis	Enhances migration of MSCs to injury sites, leading to improved angiogenesis.	15 μg/L nanoparticles of AuFe 3 μg/mL of ps-TNCs Dose not specified— 100 μg of Fe used for labeling 1 × 10^6^ MSCs	Mouse model of skin wounds; Mouse hindlimb ischemia model (angiogenesis) (used human ASCs); Rat models liver fibrosis (rat BM-MSCs)	[122,123,124]

Abbreviations: ASCs—adipose tissue mesenchymal stem cells, AT-MSCs—adipose tissue mesenchymal stem cells, AuFe – gold-iron (nanoparticles), BM-MSCs—bone marrow mesenchymal stem cells, ECM—extracellular matrix, EPC—endothelial progenitor cell, hUC-MSCs—human umbilical cord-derived mesenchymal stem cells, IONPs—iron oxide nanoparticles, LDH—lactate dehydrogenase, Mg-BCP—magnesium ion substituted biphasic calcium phosphate, MSCs—mesenchymal stem cells, PBS—phosphate-buffered saline, PLLA@MOF—Zn-Cu imidazole MOF coated poly-L-lactic acid nanofiber scaffold, SCB-MSCs—subchondral bone mesenchymal stem cells, ps-TNCs—pH-sensitive transition metal nanoclusters, TGF-β—transforming growth factor-beta, TNF-α—tumor necrosis factor-alpha, ucMSCs—umbilical cord mesenchymal stem cells, VEGF—vascular endothelial growth factor, the arrow upwards (↑) indicates stimulation or an increase, the arrow downwards (↓) indicates inhibition or a decrease.

## 7. Modulation of MSC Immunoregulatory Functions by Vitamins and Minerals

Mesenchymal stem cells possess profound immunomodulatory capabilities that are critical for their therapeutic potential in inflammatory and autoimmune diseases. These functions are not static but can be modulated by microenvironmental factors, including vitamins C, D, A and B6, along with minerals like magnesium, zinc, and iron which can modulate the immunosuppressive and anti-inflammatory properties of MSCs by altering their cytokine secretion profiles, surface marker expression, and intracellular signaling (Table 4).

### Immunomodulation by Micronutrients

Vitamin C contributes to immune regulation by suppressing the senescence-associated secretory phenotype (SASP) in MSCs. This action reduces the production of proinflammatory cytokines, thereby helping to maintain the functional integrity of MSCs and support the activity of immune effector cells [115,129]. Vitamin D exerts potent immunomodulatory effects by reducing the secretion of proinflammatory cytokines from MSCs, a process mediated by the inhibition of NF-κB signaling [42,130,131,132]. This mechanism also enhances the ability of MSCs to suppress T cell proliferation, further strengthening their immunosuppressive capacity [130,133,134]. Vitamin B6 has been shown to modulate the immunogenic and immunoregulatory profile of MSCs by increasing the surface expression of HLA molecules while also upregulating the immunomodulatory molecule PD-L1 and the anti-inflammatory factor IL-1RA [120]. Retinoic acid (RA) can enhance the ability of MSCs to suppress T cell proliferation and acts synergistically with vitamin B6 to boost PD-L1 expression [120]. However, in other contexts, RA can also activate pro-inflammatory pathways within MSCs [135].

The anti-inflammatory properties of magnesium are well-documented. It significantly reduces the production of proinflammatory cytokines by MSCs while simultaneously increasing levels of anti-inflammatory mediators like IL-10 and PGE2. This effect is associated with the modulation of key inflammation-related transcription factors, including NF-κB, thereby enhancing the overall immunosuppressive function of MSCs [136]. Zinc is also essential for regulating the immune response, as its deficiency is linked to enhanced inflammation. Conversely, zinc supplementation exerts anti-inflammatory effects and modulates the expression of genes involved in key inflammatory signaling pathways in MSCs [137]. It was found, MSCs treated with iron delivered to cells as oxide nanoparticles (IONPs) exhibit enhanced anti-inflammatory properties, characterized by a shift in their cytokine profile toward reduced secretion of proinflammatory factors and increased levels of anti-inflammatory cytokines [138].

**Table 4:** Modulation of MSC immunoregulatory functions by vitamins and minerals.

Substance	Effect	Mechanism	Concentration	Cell Type/Model	Refs.
**Vitamin C**	↓ Reduces pro-inflammatory phenotype	Suppresses Senescence-Associated Secretory Phenotype (SASP), reducing TNF-α, IL-1β, IL-6, IL-8. Supports activation of NK cells and cytotoxic T lymphocytes.	200 µmol/L	SCS-MSCs (cells with prelamin A overexpression—MSC/PLA)	[115]
**Vitamin D**	↓ Strong anti-inflammatory effects	Reduces secretion of TNF-α, IL-1β, IL-6 by blocking NF-κB signaling. Enhances MSC-mediated suppression of CD4+ T cell proliferation. Suppresses M1 macrophage differentiation. Promotes M2 macrophage differentiation. Inhibits M1 macrophage-mediated MSC migration in a dose-dependent manner.	100 nM 1.25(OH)_2_D_3_ 10 nM and 100 nM 100 and 1000 ng/kg, s.c. detrimental effect during proinflammatory stage, neutral effect during regenerative phase 15,000 IU/kg b.w. 3000 IU/kg diet	Human periodontal ligament stem cells (hPDLSCs); **In vitro**: mouse BM-MSCs; **In vivo***:* studies with mice (bone fracture healing); **In vivo**: mouse acute model, LPS; **In vivo**: mouse chronic model, HF diet	[42,130,132]
**Vitamin B6**	↑ Enhances immunogenicity and immunomodulatory potential	Increases surface expression of HLA classes I and II. Upregulates PD-L1 and markedly increases IL-1RA mRNA levels.	1 µM	Human umbilical cord-derived MSCs (ucMSCs)	[120]
**Retinoic Acid**	↑ Enhances immunosuppression (but can be pro-inflammatory)	Suppresses CD4+ and CD8+ T cell proliferation. Decrease PD-L1 expression (with Vit B6). Significantly increase HLA class I and HLA class II expression. Reduces TNF-α secretion. In another context, it activates the pro-inflammatory NF-κB/NLRP3 axis: promotes the production of the proinflammatory cytokine interleukin-1β.	1 µM or 10 µM (effect achieved when RA was part of the MC cocktail) ucMSCs pre-treated with 1 µM or 10 µM RA—no effect 1 µM or 10 µM—limited effect (only TNF-α inhibition) 100 nM, 1 µM, 10 µM (proinflammatory effect)	**In vitro**: human umbilical cord-derived MSCs (ucMSCs) **In vivo**: liver disease mouse model **Ex vivo**: liver inflammation co-culture model Human periodontal ligament stem cells (hPDLSCs)	[120,135]
**Magnesium**	↓ Strong anti-inflammatory and immunosuppressive effects	Reduces IL-1β, IL-6; increases IL-10, PGE2. Modulates NF-κB and STAT3.	5 mM	Murine MSCs; Macrophages (via conditioned medium)	[136]
**Zinc**	↓ Exerts anti-inflammatory effects	Upregulates genes for cytokine receptor interactions and IL-17/TNF pathways. Deficiency increases TNF-α, IL-1β, IL-8.	5 µmol/L	Human umbilical cord-derived MSCs (hUC-MSCs)	[137]
**Iron (IONPs)**	↓ Enhances anti-inflammatory properties	Shifts cytokine profile to reduce pro-inflammatory factors (IL-2, TNF-α) and increase secretion of anti-inflammatory IL-4, IL-10) cytokines.	50 μg/mL of Fe3O4@PDA nanoparticles 1 × 10^6^ MSCs labeled with Fe3O4@PDA nanoparticles	**In vitro**: rat BM-MSCs **In vivo**: laser burn wound rat model	[138]

Abbreviations: BM-MSCs—bone marrow stromal cells, CD4+ T cell—CD4 positive T cell, CD8+ T cell—CD8 positive T cell, Fe3O4@PDA-polydopamine-coated magnetite, HF—high-fat (diet), HLA—human leukocyte antigen, hPDLSCs—periodontal ligament stem cells, hUC-MSCs—human umbilical cord-derived mesenchymal stem cells, IL-1—interleukin-1, IL-10—interleukin-10, IL-17—interleukin-17, IL-1RA—interleukin-1 receptor antagonist, IL-1β—interleukin-1 beta, IL-4—interleukin-4, IL-6—interleukin-6, IL-8—interleukin-8, IONPs—iron oxide nanoparticles, LPS—lipopolysaccharide, MC—multiple cytokine combination (IFN-γ, TGFβ, retinoic acid), MSC—mesenchymal stem cell, mRNA—messenger ribonucleic acid, NF-κB—nuclear factor kappa-light-chain-enhancer of activated B cells, NK cells—natural killer cells, NLRP3—NLR family pyrin domain containing 3, PD-L1—programmed death-ligand 1, PGE2—prostaglandin E2, PLA–prelamin A, RA—retinoic acid, SASP—senescence-associated secretory phenotype, SCB-MSCs—subchondral bone mesenchymal stem cells, STAT3—signal transducer and activator of transcription 3, TNF—tumor necrosis factor, TNF-α—tumor necrosis factor-alpha, ucMSCs—umbilical cord-derived mesenchymal stem cells, the arrow upwards (↑) indicates stimulation or an increase, the arrow downwards (↓) indicates inhibition or a decrease.

## 8. Antioxidant Regulation and Redox Homeostasis in MSC Biology

The therapeutic efficacy of mesenchymal stem cells is intrinsically linked to the maintenance of redox homeostasis. Oxidative stress, resulting from an imbalance between reactive oxygen species (ROS) and antioxidant defense systems, impairs mitochondrial function, accelerates senescence, and compromises the regenerative capacity of MSCs [55,139,140]. Consequently, MSCs rely on antioxidant enzymes, supported by both endogenous mechanisms (e.g., glutathione regeneration) and exogenous factors. In particular, vitamins and minerals protect against cellular damage by several mechanisms, on one hand, by reducing intracellular ROS levels and, on the other hand, by activating multiple protective signaling pathways, as shown in Table 5 [61,77,141,142,143,144].

### The Role of Vitamins in Redox Regulation

As a potent antioxidant, vitamin C directly scavenges ROS and supports mitochondrial homeostasis, thereby protecting MSCs from oxidative damage and resulting enhanced senescence. It also exerts its protective effects by inhibiting ROS production through key signaling pathways (Table 5) [14,32,55,77,125,145]. Vitamin D contributes to redox balance by activating the vitamin D receptor (VDR), which leads to the upregulation of antioxidant enzymes. This mechanism helps preserve mitochondrial function, reduce ROS accumulation, and protects MSCs from oxidative injury [59,61]. It can also promote autophagy and enhance resistance to oxidative stress, especially when combined with other metabolic modulators [55,146,147,148,149]. As a major fat-soluble antioxidant, vitamin E is crucial for protecting cell membranes from lipid peroxidation, thereby shielding the MSC lipid bilayer from oxidative stress and exerting anti-aging protection [77,82,150]. This helps maintain MSC viability and function, particularly under the challenging conditions found in damaged tissues.

Trace elements are essential for antioxidant defense. Selenium, particularly in nanoparticle form, exerts a potent, dose-dependent protective effect against oxidative stress (Table 5). At optimal concentrations, it reduces intracellular ROS and activates signaling pathways that increase the expression of key antioxidant enzymes, thereby improving MSCs viability [49]. Zinc enhances cellular defense by modulating pathways that promote the transcription of antioxidant genes [137,151]. Iron exhibits a dualistic role, while low levels can stimulate adaptive repair mechanisms, excessive iron can lead to toxic ROS accumulation, underscoring the importance of tightly regulated iron homeostasis [122,152,153,154].

**Table 5:** The micronutrients in antioxidant regulation and redox homeostasis in MSC biology.

Substance	Effect/Role	Mechanism	Concentration	Cell Type/Model	Refs.
**Vitamin C**	Potent antioxidant, reduces ROS	Scavenges ROS; inhibits ROS production via AKT/mTOR axis.	200 µmol/L	Adipose-tissue MSCs	[14,32,55,125,155]
**Vitamin D**	Maintains redox balance	Activates VDR to upregulate SOD2. Preserves mitochondrial function.	100 nmol/L 1,25(OH)_2_D (ROS reduction model); 1–100 nM (SOD2 dose–response) 0.1 µg/kg 1,25(OH)_2_D i.p.	**In vitro**: human and mouse BM-MSCs **In vivo***:* HFD-induced osteoporosis model in mice	[59,61]
**Vitamin E**	Protects from oxidative stress	Prevents lipid peroxidation of cell membranes. Reduces oxidative stress and aging caused by hydrogen peroxide (H_2_O_2_). Maintenance of cell membrane integrity. Protects against apoptosis and promotes survival, enhances proliferation.	50 µM 500 µM 50 µM and 100 µM (in vitro) Rat BM-MSCs pretreated with 100 µM	**In vitro**: Human DPSCs; porcine ASCs; rat BM-MSCs **In vivo**: osteoarthritis (OA) rat model	[77,78,145]
**Selenium (SeNPs)**	Protects against oxidative stress	Activates JNK/FOXO3a pathway, increasing SOD and catalase.	50 ng/mL (optimal) >100 ng/mL (cytotoxic)	hESC-derived MSCs; rBM-MSCs	[49,141,144]
**Zinc**	Enhances antioxidant defense	Modulates Nrf2/Sirt3 pathway to promote antioxidant gene transcription.	5–100 µM	Human UC-MSCs	[137,151]
**Iron**	Dualistic: adaptive vs. toxic	Low concentrations: mild ROS for adaptive response (HIF-1α stimulation). High concentrations: toxic ROS.	Adaptive: 3 μg/mL and 50 μg/mL. Toxic (high ROS/damage): >3 μg/mL; 15.4 μg/mL; >50 μg/mL	Human ADSCs; Human BM-MSCs; Rat BM-MSCs	[122,152,153,154]

Abbreviations: ADSCs, ASCs—adipose derived mesenchymal stem cells, AKT—protein kinase B, BM-MSCs—bone marrow-derived mesenchymal stem cells, DPSCs—dental pulp stem cells, FOXO3a—forkhead box O3a, hESC—human embryonic stem cell, HFD—high fat diet, HIF-1α—hypoxia-inducible factor 1 subunit alpha, i.p.— intraperitoneal, JNK—c-Jun N-terminal kinase, MSCs—mesenchymal stem cells, mTOR—mammalian target of rapamycin, Nrf2—nuclear factor erythroid 2-related factor 2, OA—osteoarthritis, ROS—reactive oxygen species, SeNPs—selenium nanoparticles, Sirt3—sirtuin 3, SOD—superoxide dismutase, SOD2—superoxide dismutase 2, UC-MSCs—human umbilical cord-derived mesenchymal stem cells, VDR—vitamin D receptor.

## 9. Epigenetic Regulation of Genomic Stability in Mesenchymal Stem Cells by Vitamins and Minerals

The genomic stability of MSCs is maintained through tightly regulated epigenetic mechanisms, including DNA methylation and histone modifications. Various micronutrients, such as vitamins A, C, and B3 as well as minerals like iron, zinc, and selenium, are crucial for this process (Table 6). They act as cofactors or modulators for key epigenetic enzymes, thereby preserving the epigenetic integrity of MSCs and enhancing their therapeutic potential [13,55,61,71,139,156,157,158]. Vitamin C is a direct epigenetic modulator, serving as an essential cofactor for both TET enzymes, which initiate active DNA demethylation, and Jumonji C-domain-containing histone demethylases, which regulate gene expression [8,159]. Its function is closely linked to iron availability, which is required for the optimal activity of these enzymes [159]. Vitamin B3 (nicotinamide) contributes to epigenetic regulation by activating SIRT1, a NAD^+^-dependent histone deacetylase that is involved in delaying senescence and maintaining telomere integrity [65].

Zinc regulates epigenetic function by activating histone deacetylases like SIRT3 and by serving as an essential structural component for numerous DNA-binding proteins, including transcription factors and chromatin remodelers. Therefore, the supplementation with zinc can support telomeric stability by influencing the expression of key genes [160]. Selenium plays an indirect yet crucial role in epigenetic maintenance by supporting the methylation cycle. As a key component of selenoproteins, it ensures the availability of S-adenosylmethionine, the universal methyl donor required for DNA and histone methylation. This function is vital for preserving chromatin integrity and reducing DNA damage in MSCs [140].

**Table 6:** The role of selected micronutrients in MSC epigenetic regulation.

Substance	Role/Effect	Mechanism	Concentration	Cell Type/Model	Refs.
**Vitamin C**	Directly modulates DNA and histone demethylation	Serves as a cofactor for TET enzymes, promoting active DNA demethylation (5mC → 5hmC). Supports Jumonji (JHDM) histone demethylases (e.g., of H3K36me2/3), leading to increased c-Myc/Klf4 and repression of p21.	200–250 µM	Gingival stem cells; mouse ASC	[8,126,159]
**Vitamin B3**	Regulates histone deacetylation and telomere maintenance	Activates SIRT1, a NAD^+^-dependent histone deacetylase, which delays senescence and supports differentiation.	5 mM	Mouse adipose-derived MSCs	[65]
**Zinc (Zn^2+^)**	Regulates histone deacetylation and telomeric stability	Activates SIRT3 (histone deacetylase). Acts as a structural component for zinc finger transcription factors and chromatin remodelers. Increases TERT expression.	0.14 µg/mL ZnSO_4_	Rat adipose-derived MSCs	[160]
**Selenium**	Supports methylation cycle and chromatin integrity	As a component of selenoproteins, it ensures the availability of S-adenosylmethionine (SAM), the universal methyl donor for DNMTs and HMTs. Reduces DNA damage indicators (micronuclei) by up to 58%.	100 nM sodium selenite	Human BM-MSCs	[140]

Abbreviations: 5hmC—5-hydroxymethylcytosine, 5mC—5-methylcytosine, ASC—adipose tissue mesenchymal stem cells, BM-MSCs—bone marrow mesenchymal stem cells, c-Myc—cellular myelocytomatosis, DNMTs—DNA methyltransferases, H3K36me2/3—histone H3 lysine 36 dimethylation/trimethylation, HMTs—histone methyltransferases, JHDM—Jumonji C domain-containing histone demethylases, Klf4—Krüppel-like factor 4, MSCs—mesenchymal stem cells, NAD^+^—nicotinamide adenine dinucleotide, p21—protein 21, SAM—S-adenosylmethionine, SIRT1—sirtuin 1, SIRT3—sirtuin 3, TERT—telomerase reverse transcriptase, TET—ten-eleven translocation.

## 10. Regulation of MSC Activity by Phytochemicals

**Figure 3:**
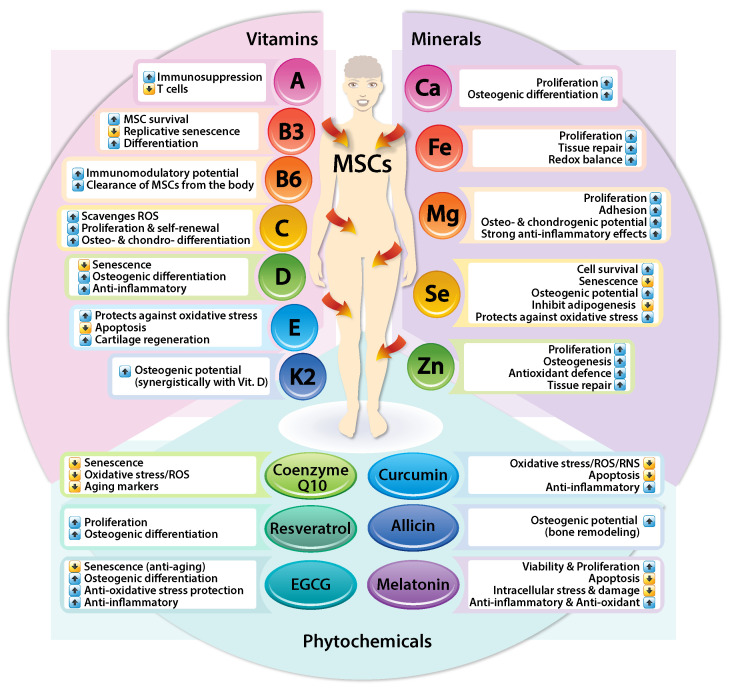
Key modulators of mesenchymal stem cell function. This schematic provides an overview of essential micronutrients, categorized as vitamins and minerals, and phytochemicals that regulate the activity of mesenchymal stem cells. Each substance plays a specific role in influencing stem cell fate and supporting the body’s regenerative processes. The collective action of these molecules is crucial for creating an optimal microenvironment to maintain the health and function of the MSC pool (the blue arrow upwards (↑) indicates stimulation or an increase, the yellow arrow downwards (↓) indicates inhibition or a decrease).

Mesenchymal stem cells, due to their multipotency, immunomodulatory properties, and paracrine activity, are a valuable therapeutic tool in the treatment of inflammatory, degenerative, and post-traumatic conditions. Nevertheless, their therapeutic potential largely depends on the local microenvironment, including biochemical, metabolic, and nutritional factors that can modulate their biological functions. With the rapid development of regenerative medicine and new strategies, there is growing interest in nutritional strategies aimed at increasing MSC activity and improving the effectiveness of cell-based therapies. Particular attention has been paid to natural phytochemicals such as curcumin, epigallocatechin gallate (EGCG), resveratrol, allicin, coenzyme Q10, and melatonin (Figure 3). The phytochemicals have antioxidant, anti-inflammatory, mitochondria-protecting, and metabolism-activating properties, and they also influence key signaling pathways related to MSC survival, differentiation, migration, and regenerative capacities [161,162,163,164,165,166,167,168,169,170,171,172,173,174,175,176].

Epigallocatechin gallate (EGCG), the main polyphenol found in green tea, is also found in fruits (cranberries, strawberries, blackberries, apples, avocados) and several nuts (pistachios). It has strong antioxidant and anti-inflammatory properties. EGCG modulates biological processes beyond basic nutritional functions. Research confirm that EGCG can have an effect on mesenchymal stem cells (MSCs) by regulating their proliferation, differentiation, and immunomodulatory properties, indicating its potential role in regenerative therapies and in the treatment of inflammatory and degenerative diseases [161,162,163]. The study conducted by Shin et al. (2016) showed that EGCG prevents hydrogen peroxide (H_2_O_2_)-induced aging of human MSCs [161]. This protective effect is mediated by the activation of the Nrf2 transcription factor, which regulates the expression of antioxidant genes, and by the downregulation of acetylated p53 and p21 proteins, which have been implicated in cellular aging. In MSCs with silenced Nrf2, EGCG did not show a protective effect, resulting in high levels of acetylated p53 and p21 despite prior administration of EGCG. This underlines the key role of the Nrf2 pathway in mediating the antioxidant and anti-aging effects of EGCG [161,162].

Similarly to EGCG, curcumin, a naturally occurring polyphenol found in the roots of *Curcuma longa*, exerts a protective effect on human mesenchymal stem cells under conditions of oxidative stress. A study conducted by Yagi et al. (2013) showed that both EGCG and curcumin inhibited the production of reactive oxygen species (ROS) and reactive nitrogen species (RNS), acting as potent modulators of inflammation [164]. In this study, an evaluation was conducted to assess the effect of polyphenols on hMSCs exposed to oxidative stress induced by hydrogen peroxide and S-nitroso-N-acetylpenicylamine (SNAP). The parameters evaluated included colony formation activity, apoptosis, antioxidant enzyme levels, and the presence of reactive oxygen species. The polyphenols reversed the H_2_O_2_-induced loss of colony formation activity in hMSCs. They dose-dependently inhibited the elevated levels of ROS and NO generated by H_2_O_2_ and SNAP, respectively. Remarkably, the polyphenols rapidly and almost completely blocked H_2_O_2_-induced ROS without directly neutralizing H_2_O_2_ itself. In addition, they preserved the function of antioxidant enzymes and reduced cell apoptosis caused by exposure to H_2_O_2_. Likewise, Wang et al. (2016) demonstrated that EGCG protects bone marrow-derived mesenchymal stem cells (BM-MSCs) from H_2_O_2_-induced inhibition of osteogenic differentiation. EGCG not only reversed the negative effects of oxidative stress, but also independently enhanced osteogenesis by upregulating β-catenin and cyclin D1, indicating the involvement of Wnt signaling. This effect was inhibited by the Wnt pathway inhibitor DKK-1, confirming the action of EGCG through Wnt modulation. Together, these findings highlight the potential of EGCG in promoting bone regeneration under oxidative conditions [165].

Allicin, a phytochemical found in garlic extract, has the potential to modulate hMSC function by influencing their differentiation into osteoblasts, which is crucial for bone regeneration and is important in bone tissue engineering and osteogenesis. As a result, allicin becomes a valuable dietary component that supports skeletal health. Bose et al. (2023) showed that garlic extract, allicin released from bioceramic calcium phosphate scaffolds, significantly enhances osteogenesis in co-cultures of hMSCs and monocytes [166]. A threefold increase in ALP (alkaline phosphatase) and a 1.6-fold increase in BGLAP (bone gamma-carboxyglutamic acid) were observed. Protein expression after 14 days indicated accelerated differentiation of osteoblasts. Elevated expression of the receptor activator of nuclear factor κB (RANKL) and transient expression of tartrate-resistant acid phosphatase (ACP5) suggest active bone remodeling, later balanced by increased expression of OPG (osteoprotegerin). The pro-osteogenic effect was corroborated by a fourfold increase in phosphatase activity and increased collagen accumulation in vivo in rats.

Resveratrol occurs naturally in many plants. Major dietary sources include the skins of red grapes (and thus red wine), blueberries, cranberries, peanuts, blackcurrants, and Japanese knotweed (*Polygonum cuspidatum*), plant widely used in traditional Chinese medicine. Resveratrol promotes the proliferation and differentiation of human mesenchymal stem cells derived from bone marrow (hBM-MSCs). In vitro studies Dai et al. (2007) have shown that resveratrol increases alkaline phosphatase (ALP) activity, calcium deposition in the extracellular matrix, and the expression of osteoblastic markers such as RUNX2, Osterix, and Osteocalcin [167]. These effects are mediated through activation of the ERK1/2 signaling pathway via estrogen receptors, as confirmed using antagonists and specific pathway inhibitors. The data suggest that RSVL stimulates osteogenesis in hBM-MSCs in an estrogen receptor- and ERK1/2-dependent manner, with additional involvement of the p38 MAPK pathway.

Coenzyme Q10 (CoQ10), also known as ubiquinone, is a lipophilic compound with a quinone structure, naturally occurring in the mitochondrial membranes of eukaryotic organisms, where it plays a key role in electron transport and protection against oxidative stress. Exogenous sources of CoQ10 mainly include animal products such as meat, offal (especially liver), and fatty fish. It is also found in plant products, including vegetable oils and nuts, but its content in vegetables, fruits, dairy products, and eggs is relatively low. CoQ10 is also present in breast milk. Studies have shown that its concentration is higher in women who have given birth to full-term babies than in mothers of premature babies [168,169,170]. The concentration of CoQ10 in breast milk decreases as lactation progresses. In vitro studies have shown that CoQ10 can counteract the aging of MSCs induced by D-galactose [172]. D-galactose increases the production of reactive oxygen species (ROS), which leads to an increase in the number of cells exhibiting SA-β-galactosidase activity and increased expression of senescence-related genes such as p53, p21, and p16. CoQ10 supplementation significantly reduced ROS levels and the expression of these aging markers. In addition, CoQ10 was observed to affect the expression of genes involved in MSC adipogenic differentiation (e.g., PPARγ) and genes encoding antioxidant enzymes, suggesting its ability to modulate the phenotype and metabolic activity of these cells. Mechanistically, the anti-aging effect of CoQ10 may be mediated by inhibition of the Akt/mTOR signaling pathway, which plays a central role in the regulation of cellular homeostasis and senescence processes [173].

Another important phytochemical is melatonin (MLT). MLT is an endogenous indoleamine compound with a broad spectrum of biological activity, also found in various plants and animal foods. Among plant sources of melatonin, nuts (especially walnuts) deserve special attention, as they contain the highest amounts. Melatonin is also present in fruits (e.g., mangoes, pineapples, kiwis, white mulberries), vegetables (e.g., peppers), grains, some mushrooms, as well as in sprouting seeds and legumes. Among animal products, higher concentrations of melatonin are found in eggs and fish [174]. In addition to regulating the circadian rhythm, MLT has metabolism-regulating properties and acts as an anti-inflammatory and antioxidant agent. In recent years, there has been growing interest in the effects of melatonin on mesenchymal stem cells, not only regarding their survival, but also their paracrine signaling activity and secretion of extracellular vehicles (EVs) [175]. Li B et al. (2021) reported that MLT stimulates the viability of adipose-derived mesenchymal stem cells (ADMSCs) by upregulating the TGF-β pathway and decreasing ER stress in metabolic disease models [176]. MLT enhanced ADMSC proliferation, reduced ER stress signals, and increased their therapeutic capacity in type 2 diabetes mellitus (T2DM) models (pancreatic regeneration, reducing inflammatory and metabolic stress). Melatonin at 0–10 μM during 24 h incubation can effectively alleviate excessive apoptosis and mitochondrial dysfunction in mesenchymal stem cells derived from the nucleus pulposus induced by oxidative stress via the PI3K/Akt pathway, which may provide a new concept for the treatment of intervertebral disc degeneration [171].

As stimulators, phytochemicals significantly influence proliferation and differentiation and accurately regulate MSCs via a variety of protein pathways. In an addition, they exhibit fewer toxic effects, are cost-effective, and have the potential to enhance the efficacy of cell therapies using MSCs for both non-illnesses and communicable diseases. Moreover, phytochemicals may exert synergistic or additive effects in cells. The regular intake of phytochemicals such as curcumin, EGCG, resveratrol, allicin, coenzyme Q10, and melatonin might significantly enhance the efficacy of regenerative therapies involving MSCs. Curcumin and EGCG, as well as curcumin and coenzyme Q10, have shown potential to improve the resilience of MSCs to oxidative stress, chronic inflammation, and tissue injury [177,178,179]. Curcumin and resveratrol modulate the expression of genes involved in antioxidant defense and inflammatory signaling [180,181,182]. On the other hand, EGCG and allicin contribute to mitochondrial stabilization by limiting the excessive production of ROS [179,183]. Evaluating dietary patterns and adjusting nutrition to increase bioactive phytochemical intake should be considered an integral part of therapeutic planning, especially for regenerative strategies targeting bone, cardiovascular, and skeletal muscle tissues [184]. The detailed mechanisms underlying the regulation of mesenchymal stem cell activity by plant-derived phytochemicals are summarized in Table 7, which provides an overview of their biological effects and molecular pathways involved.

**Table 7:** Regulations of MSC activity by plant-derived phytochemicals.

Substance	Effect	Mechanism	Concentration	Cell Type/Model	Refs.
**Epigallocatechin gallate** (EGCG)—polyphenol (green tea)	Protects against oxidative stress and cell aging, and increases proliferation and osteogenesis	Activation of the Nrf2 pathway, decrease in p53/p21 acetylation; enhancement of Wnt/β-catenin, increase in cyclin D1	10–50 µM	hMSC, BM-MSC (human)	[161,162,165]
**Curcumin**— polyphenol (Curcuma longa)	Protects MSCs from oxidative stress, reduces apoptosis, supports differentiation	Inhibition of ROS and NO production, maintenance of antioxidant enzyme activity	5–20 µM	hMSCs (human)	[164]
**Allicin**— an organosulfur compound found in garlic	Enhance osteogenesis and bone remodeling	Increases expression of ALP, BGLAP, RANKL, OPG; activates osteoblast cells. Allicin released from bioceramic scaffolds (amount depends on the material).	Allicin released from bioceramic scaffolds (amount depends on the material)	Co-culture of hMSCs + monocytes, in vivo, rat model	[166]
**Resveratrol**— polyphenol (grapes, peanuts, blueberries)	Increases osteoblast proliferation and differentiation	Activation of the ERK1/2 pathway via the estrogen receptor; involvement of p38 MAPK.	1–10 µM	hBMSC (human)	[167]
**Coenzyme Q10** (ubiquinone)—lipophilic quinone	Inhibits MSC aging, reduces oxidative stress and expression of aging genes (p53, p21, p16)	Reduction of ROS, inhibition of the Akt/mTOR pathway, regulation of PPARγ expression and antioxidant enzymes.	1–100µM (D-galactose-induced aging)	hMSC (human)	[172]
**Melatonin**— indoleamine (endogenous, plant-derived)	Increases survival, proliferation, and paracrine activity of MSCs, reduces ER and mitochondrial stress	TGF-β activation, PI3K/Akt, antioxidant and anti-inflammatory effects	0–10 µM (24 h)	ADMSC, NPMSC (human)	[171,175,176]

Abbreviations: ADMSC—Adipose-derived mesenchymal stem cells, Akt—AKT serine/threonine kinase 1, ALP—alkaline phosphatase, BGLAP—bone gamma-carboxyglutamate protein, BM-MSC—bone marrow-derived mesenchymal stem cells, ERK1/2—extracellular signal-regulated kinases, hBMSC—human bone marrow-derived mesenchymal stem cells, hMSC—human mesenchymal stem cells, MAPK—mitogen-activated protein kinases, mTOR—mechanistic target of rapamycin, NO—nitric oxide, NPMSC—nucleus pulposus-derived mesenchymal stem cells, Nrf2—nuclear factor erythroid 2–related factor 2, OPG—osteoprotegerin, PI3K—phosphoinositide 3-kinase, PPARγ—peroxisome proliferator-activated receptor gamma, RANKL—receptor activator of nuclear factor κB ligand, ROS—reactive oxygen species, TGF-β—transforming growth factor-beta, Wnt/β-catenin—wingless/integrated/beta-catenin.

## 11. Critical Assessment and Translational Challenges in the Application of Bioactive Compounds for Mesenchymal Stem Cell Therapy

Recent studies underscore the importance of adequate and balanced nutrient intake in strategies aiming at optimizing the therapeutic potential of MSCs. More broadly, the integration of biochemical signals derived from vitamins, minerals, and other bioactive compounds forms a complex regulatory network that governs stem cell behavior across adult stem cells lineages, including mesenchymal stem cells. A deeper understanding of these molecular mechanisms is critical for translating fundamental insights into clinical applications.

Vitamins and mineral components are integral constituents of stem cell growth conditions. Additionally, selected secondary metabolites derived from plants may also elicit specific biological effects on stem cells (Figure 3). Finally, the precise influence of vitamins and minerals on MSCs underscores their potential for optimized preconditioning strategies aimed at maximizing therapeutic performance in regenerative medicine. Food fortification is a practical and clinically relevant strategy for improving patients’ nutritional status and facilitating regenerative medicine research. The practice of voluntarily fortifying commonly consumed foods can reduce the population-level risk of suboptimal micronutrient intake while improving intake and biochemical status of key micronutrients, including folate, vitamin D, and riboflavin, in both children and adults [185]. In Austria, fortified products significantly contribute to daily micronutrient intake without the risk of excessive consumption, demonstrating the safety and effectiveness of this approach [186]. Maintaining optimal micronutrient status is crucial. While the direct clinical evidence linking micronutrient concentrations to the efficacy of MSC therapy is scarce, data from hematopoietic stem cell transplantation indicate that common vitamin and mineral deficiencies may influence recovery, immune function, and worsen clinical outcomes in patients [187,188]. These findings underscore the potential role of fortified foods and personalized nutritional strategies to support micronutrient availability in humans and create a favorable environment for MSC survival, proliferation, and differentiation.

Although promising findings have been obtained from cellular and animal models, they cannot be directly extrapolated to human physiology. Therefore, there is a pressing need for comprehensive clinical studies in humans to validate these mechanisms in vivo. Furthermore, these studies must also establish precise relationships between specific doses of vitamins or minerals and their biological effects, considering the context of bioavailability for these bioactive compounds. Such detailed knowledge would be instrumental in the targeted design of functional foods aimed at modulating specific stem cell populations in vivo. A key translational challenge is the fact that promising in vitro results do not always translate to in vivo success. Many studies are exclusively in vitro and require in vivo validation. For example, while iron particles stimulated MSC proliferation and differentiation in vitro, the resulting in vivo bone formation was not significantly altered [189]. The benefits of confirming the results of in vitro studies on the effect of minerals on MSCs in in vivo models would be obvious, especially in cases where the mineral has a positive effect on physiological processes through several mechanisms. Such a study should be performed to find out if magnesium may enhance chondrogenic differentiation by inhibiting macrophage-induced inflammation in humans [190]. Similarly, the study showing that elevated extracellular calcium (6–10 mM) promotes MSC proliferation and migration was done in vitro only, and the results require further in vivo investigation [80].

A recurring gap is the lack of reliable data on optimal concentrations and combinations for many bioactive compounds that modulate MSCs function in vivo [191]. We can note significant obstacles in determining the appropriate dose, the risk of adverse effects (including tumorigenicity) and the need for standardized safety studies [118]. In particular, it should be emphasized that MSCs exhibit significant heterogeneity depending on their tissue origin [191]. This is a critical gap in current knowledge, as a preconditioning strategy may not be universally effective. For instance, resveratrol at 0.1 µM promotes proliferation, yet 5 µM inhibits self-renewal and increases senescence [7]. The in vivo efficacy of vitamin C priming was found for hES-MSCs and hUC-MSCs, but its efficacy for hBM-MSCs was not confirmed in that study [192]. Furthermore, many researchers (e.g., [14,70,193,194]) explicitly point out a lack of information on the impact of the donor’s or recipient’s nutritional status on therapeutic outcomes. There is a distinct lack of direct data correlating dietary components or the intake of vitamins and minerals with the quality and quantity of MSCs from a given patient or donor.

However, the findings presented in this review, even with limitations, carry significant clinical implications for clinical use of MSCs, especially in regenerative medicine. For example, several approaches target bone and cartilage repair directly. Zinc supplementation is a promising support for pharmaceutical therapies for osteoporosis and other bone loss conditions [195]. In parallel, Vitamin E shows potential for improving cartilage repair in conditions like osteoarthritis [118,191]. Beyond its direct repair ability, Vitamin C can enhance the immunomodulatory functions of MSCs with direct clinical relevance for inflammatory and autoimmune disorders treatment (e.g., GVHD and asthma) [192]. Similarly, magnesium’s capacity to inhibit macrophage-induced inflammation suggests its potential use in inflammatory joint diseases [190]. These elements are also central to developing tissue engineering solutions, where biomaterials incorporating minerals like zinc [193], magnesium [70,190] and calcium [80] are pivotal.

When planning the use of MSCs in regenerative medicine, it should be considered that optimizing the ex vivo expansion phase of MSCs is clinically significant. The ability of several micronutrients to enhance proliferation is crucial during that process. The addition of Vitamin C at 0.1–3 mM promotes MSC proliferation and leads to more efficient cell preparation [196]. Moreover, Vitamin C helps maintain the “stemness” or plasticity of MSCs and prevents premature senescence during culture by modulating the epigenome (e.g., TET enzyme) [14].

To sum up, currently the biggest challenge is the validation in vivo of promising in vitro findings. This includes translating observations of complex processes like the anti-inflammatory effects into relevant preclinical animal models. To support this, future work must also focus on standardization and optimization, developing reliable platforms to assess the safety and efficacy of MSCs preconditioning while determining optimal doses and combinations [118,191]. However, obtaining deeper mechanistic insights is also essential. For mineral-based therapies, the design of novel biomaterials that offer controlled degradation and ion release, is crucial [70]. This must be coupled with further investigation to elucidate the detailed molecular mechanisms, including the identification of significant signaling pathways involved in the process [70].

Finally, the research needs to focus on humans. The great challenge is to tailor particular strategies for specific diseases and cell therapies, rather than using a one-size-fits-all approach. To achieve this goal, the problems of bioavailability of particular micronutrients should be addressed. For example, novel strategies to enhance absorption should be developed. Functional foods offer a wide range of opportunities in this area. Simultaneously using a micronutrient and a specific ingredient that improves its absorption would be a perfect example of such an approach.

At the same time, it should be emphasized that micronutrients may lead to adverse health effects if consumed in excess. The European Food Safety Authority (EFSA) [197] confirmed that the tolerable upper intake level (UL) for vitamin A (retinol) is 3000 micrograms (µg) retinol equivalent (RE)/day for adults, including pregnant and lactating women. The critical toxic effect is teratogenicity—excess vitamin A can lead to fetal abnormalities. EFSA has deemed β-carotene from food safe, but no UL has been established for -carotene due to a lack of dose–response data. In particular, high doses of β-carotene found in supplements may increase the risk of lung cancer in smokers and are therefore not recommended for this population [198]. However, vitamin A derivatives, particularly all-trans retinoic acid (ATRA), have emerged as some of the most thoroughly studied agents in the context of targeting cancer stem cells (CSC). ATRA primarily exerts its anti-CSC effects by inducing differentiation in stem-like cancer cells, effectively pushing them toward terminal maturation [199,200]. This loss of stemness translates into reduced tumor-initiating capacity and increased susceptibility to conventional chemotherapies. Preclinical models, including glioblastoma [201], hepatocellular carcinoma (HCC) [202], osteosarcoma [203], and gastric cancer [204], consistently demonstrate that ATRA not only induces differentiation but also disrupts self-renewal pathways and suppresses the expression of critical CSC markers [205,206,207]. In vivo validation in xenograft and orthotopic mouse models confirms its therapeutic relevance in reducing tumor initiation and CSC-related phenotypes [200]. However, more studies focused on off-target effects should be performed in relation to doses used in experiments.

A deeper understanding of the nutritional modulation of stem cell biology holds promising translational potential for the development of targeted dietary strategies to enhance regenerative capacity, prevent stem cell exhaustion, and improve outcomes in regenerative medicine, age-related diseases, chronic health conditions, and even for cancer stem cell inhibition. In future studies, the impact of controlled nutritional interventions on the outcomes of MSC-based therapies should be systematically investigated. In these evaluations, the importance of both food intake and biochemical markers of micronutrients should be considered. Furthermore, clinical research should aim to determine the optimal dosage ranges, modes of administration and recognize potential interactions between selected micronutrients and phytochemicals. Questions arise: how can nutrition support regenerative therapies? How can nutrition enable a more complete utilization of the bioactive potential of phytochemicals? The protocols should be developed to modulate MSC function under both physiological and pathological conditions. Consequently, future studies should form a solid basis for the development of more evidence-based clinical practices.

## 12. Conclusions

Nutritional interventions specifically targeting the enhancement of stem cell functionality hold considerable promise, especially in the context of mitigating age-associated physiological decline (i.e., promoting healthy aging), as well as in the therapeutic management of chronic inflammatory conditions, osteoporosis, and neurodegenerative disorders. Recent studies suggest that these alterations may be, at least in part, modifiable through dietary strategies that influence proper epigenetic regulation, redox–inflammatory pathways and cellular metabolism. However, realization of the full potential of such strategies necessitates rigorously designed translational and clinical studies to elucidate their efficacy in vivo. Unfortunately, the limited number of advanced human studies makes it difficult to develop evidence-based, reliable nutritional strategies. The incorporation of specifically targeted functional foods as adjunctive agents in therapeutic approaches aimed at supporting tissue regeneration and maintaining cellular homeostasis represents a promising, yet underexplored, avenue.

## Data Availability

Data sharing is not applicable to this article as no datasets were generated.

## Abbreviations

The following abbreviations are used in this manuscript:

ALP	Alkaline phosphatase
BMP2	Bone morphogenetic protein 2
CaMKII	Calcium/calmodulin-dependent protein kinase II
CD	Cluster of differentiation
COL1A1	Collagen type I alpha 1 chain
ECM	Extracellular matrix
EGCG	Epigallocatechin gallate
ESC	Embryonic stem cell
hBM-MSCs	Human bone marrow-derived mesenchymal stem cells
hESC	Human embryonic stem cell
hUC-MSCs	Human umbilical cord-derived mesenchymal stem cells
hUM-MSCs	Human umbilical cord matrix-derived mesenchymal stem cells
IL-1RA	Interleukin-1 receptor antagonist
IONPs	Iron oxide nanoparticles
LDH	Lactate dehydrogenase
MSC	Mesenchymal stem cell
NF-κB	Nuclear factor kappa-light-chain-enhancer of activated B cells
Notch	Notch signaling pathway
Nrf2	Nuclear factor erythroid 2-related factor 2
OCN	Osteocalcin
PD-L1	Programmed death-ligand 1
PGE2	Prostaglandin E2
PPARγ2	Peroxisome proliferator-activated receptor gamma 2
RA	Retinoic acid
ROS	Reactive oxygen species
RSVL	Resveratrol
RUNX2	Runt-related transcription factor 2
SIRT1	Sirtuin 1
SIRT3	Sirtuin 3
STAT3	Signal transducer and activator of transcription 3
TET	Ten-eleven translocation
TGF-β	Transforming growth factor-beta
TNF-α	Tumor necrosis factor-alpha
VDR	Vitamin D receptor
VEGF	Vascular endothelial growth factor
Wnt	Wnt signaling pathway
